# Transcriptomic analysis of stage 1 versus advanced adult granulosa cell tumors

**DOI:** 10.18632/oncotarget.7422

**Published:** 2016-02-16

**Authors:** Maria Alexiadis, Simon Chu, Dilys Leung, Jodee A. Gould, Tom Jobling, Peter J. Fuller

**Affiliations:** ^1^ Hudson Institute of Medical Research (formerly Prince Henry's Institute of Medical Research), Clayton, Victoria 3168, Australia; ^2^ Monash University Department of Biochemistry and Molecular Biology, Clayton, Victoria 3168, Australia; ^3^ MHTP Medical Genomics Facility, Clayton, Victoria 3168, Australia; ^4^ Department of Gynecology Oncology, Monash Health, Clayton, Victoria 3168, Australia

**Keywords:** granulosa cells, FOXL2, ovary, transcriptome, stromal tumors

## Abstract

Ovarian granulosa cell tumors (GCT) are hormonally-active neoplasms characterized, in the adult-subtype, by a mutation in the FOXL2 gene (C134W). They exhibit an indolent course with an unexplained propensity for late recurrence; ∼80% of patients with aggressive, advanced stage tumors die from their disease; aside from surgery, therapeutic options are limited. To identify the molecular basis of advanced stage disease we have used whole transcriptome analysis of FOXL2 C134W mutation positive adult (a)GCT to identify genes that are differentially expressed between early (stage 1) and advanced (stage 3) aGCT. Transcriptome profiles for early (*n* = 6) and stage 3 (*n* = 6) aGCT, and for the aGCT-derived KGN, cell line identified 24 genes whose expression significantly differs between the early and stage 3 aGCT. Of these, 16 were more abundantly expressed in the stage 3 aGCT and 8 were higher in the stage 1 tumors. These changes were further examined for the genes which showed the greatest fold change: the cytokine CXCL14, microfibrillar-associated protein 5, insulin-like 3 and desmin. Gene Set Enrichment Analysis identified overexpression of genes on chromosome 7p15 which includes the homeobox A gene locus. The analysis therefore identifies a small number of genes with clearly discriminate patterns of expression arguing that the clinicopathological-derived distinction of the tumor stage is robust, whilst confirming the relative homogeneity of expression for many genes across the cohort and hence of aGCT. The expression profiles do however identify several overexpressed genes in both stage 1 and/or stage 3 aGCT which warrant further study as possible therapeutic targets.

## INTRODUCTION

Granulosa cell tumors of the ovary (GCT), the major form of ovarian stromal tumors, arise from proliferating granulosa cells of the ovarian follicle [1]. They exhibit features of granulosa cells that include estrogen biosynthesis as well as the production of gonadal peptides including inhibin and anti-Müllerian hormone (AMH). GCT are classified as adult (95% of GCT) or juvenile, based on histopathological and clinical criteria. The identification of a specific somatic missense mutation in the FOXL2 gene (c.402 C→G; pC134W) in ∼ 97% of aGCT [2, 3] argues strongly that this mutation both defines the disease and indeed is etiologic in the disease. A striking feature of aGCT is their propensity for late recurrence, sometimes decades after their initial identification. Although the majority of aGCT are stage 1 and are cured by surgery, approximately 80% of the patients with aggressive disease at diagnosis and/or recurrence will succumb to their disease [1]. All aGCT, whether stage 1 or advanced stage, contain the FOXL2 mutation so other genetic changes in the tumor are likely to be responsible for these differing stages and/or behaviour. The identification of molecular markers that predict recurrence and/or aggressive behaviour would be a great asset in the management of aGCT. Additionally, understanding the pathogenesis of advanced stage disease might aid the development of targeted therapies. Currently treatment options, once surgery is no longer relevant, are limited [1]. Although there have been a number of studies which explore the role of various mitogenic signaling pathways in aGCT [4–7] the specific question of what differentiates stage 1 disease from advanced stage disease has not been explored.

One approach to defining the molecular difference between stage 1 disease and advanced stage disease is to analyse their pattern of gene expression seeking critical differences and gene signatures of prognostic or therapeutic significance. There have been, until recently, relatively few whole transcriptome gene expression studies for aGCT [8]. Benayoun et al. [9] used gene expression microarrays to compare 10 GCT of mixed stage (9 × stage 1 and 1 × advanced) with two granulosa cell samples collected during IVF. In a subsequent study they correlated these findings with the results of comparative genomic hybridization (CGH) in these tumors [10]. Rosairo et al. [11] have explored gene expression in two human GCT-derived cell lines, COV434 and KGN. In the present study we have sought to identify changes in gene expression in aGCT (defined by the presence of the FOXL2 C134W mutation) that reflect the transition from stage 1 disease restricted to the ovary and therefore hopefully cured by surgery to stage 3 disease with transcoelomic spread to distant sites in the peritoneal cavity. We present the analysis of the transcription profiles for 6 stage 1 and 6 stage 3 aGCT and identify 24 genes whose expression significantly differs between the stage 1 and stage 3 GCT.

## RESULTS

The aGCT samples were obtained as previously described [3, 6]; their clinical details are in Table 1. All cases are heterozygous for the FOXL2 C134W mutation [3]. Transcription profiles for the 12 tumors and also for the KGN cell line, which is also heterozygous for the FOXL2 C134W mutation and thus derived from an aGCT [3] were obtained.

**Table 1:** Clinical information for the aGCT studied

Sample	Stage	Surgery	Menopausal Status	Age at Surgery
1*^	1	Primary	Pre	53
2*^	1	Primary	Pre	54
3*^	1c	Primary	Post	50
4*	1	Primary	Post	79
5*^	1	Primary	Pre	31
6*	1a	Primary	Pre	43
7^	1a	Primary	Post	61
8^	1	Primary	Pre	29
9*^	3	Secondary	Post	58
10*^	3	Secondary	Pre	45
11*^	3	Secondary	Post	56
12*	3	Secondary	Post	54
13*^	3	Secondary	Pre	48
14*^	3	Secondary	Post	84
15^	3	Secondary	NA	47
16^	3	Secondary	Post	70

* Microarray

^ qRT-PCR

**Figure 1:**
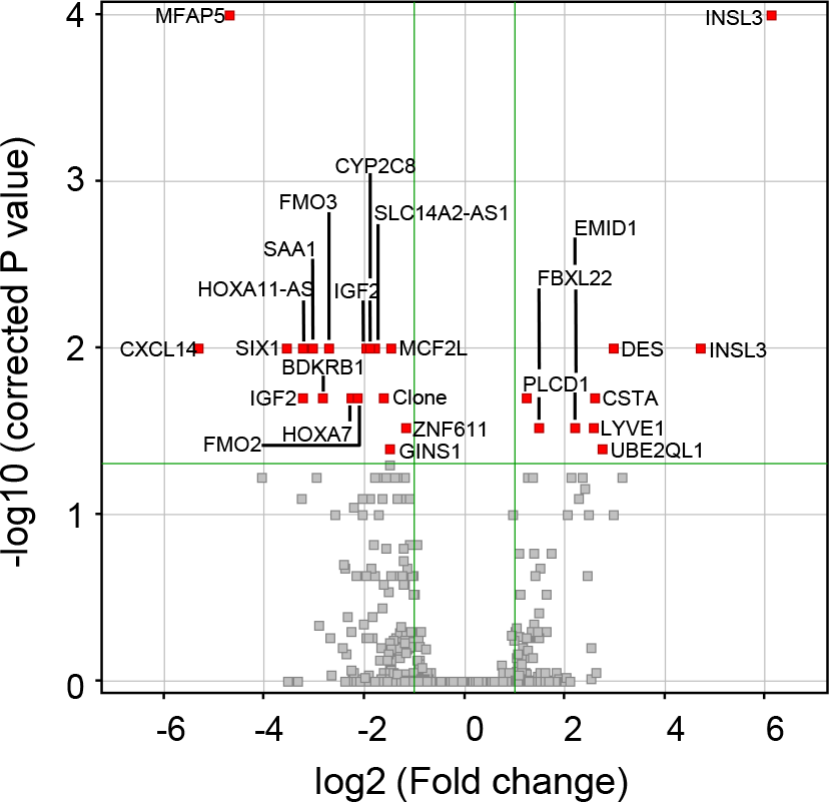
Volcano plot revealing the 26 statistically significant probes between stage 1 GCT and stage 3 aGCT representing 24 genes A moderated *t*-test was performed; the 26 probes with a *p*-value of ≤ 0.05 and ≥ 2 fold change that passed a Westfall Young Permutative multiple correction test can be seen in red with gene symbols (Table 2) indicated.

Of ∼9,000 expressed genes in both stage 1 and stage 3 aGCT, we identified 24 genes whose expression differed between the two groups. The analysis is presented as a volcano plot with the 24 genes that differ by ≥ 2-fold at a *p*-value of ≤ 0.05 and passed a Westfall Young Permutative multiple correction test represented by the 26 red points (Figure 1): 2 of the genes are represented by two independent probes on the array (Table 2). 16 genes were expressed at 2-fold higher levels in the stage 3 aGCT when compared to the stage 1 aGCT while the expression of 8 genes was down regulated in the advanced aGCT at a significance of *p* < 0.05. The full list of 26 gene probes is shown in Supplementary Table 1.

**Table 2:** Differentially expressed genes

GeneSymbol	Gene Name	FC (abs)	*p* (Corr)
CXCL14	Chemokine (C-X-C motif) ligand 14	39.933685	0.01010101
MFAP5	Microfibrillar associated protein 5, transcript variant 1	26.172625	0
SIX1	SIX homeobox 1	11.79884	0.01010101
HOXA11-AS	HOXA11 antisense RNA	9.483171	0.01010101
IGF2	Insulin-like growth factor 2, transcript variant 1	9.4279175	0.02020202
SAA1	Serum amyloid A1, transcript variant 1	8.28538	0.01010101
BDKRB1	Bradykinin receptor B1	7.1152983	0.02020202
FMO3	Flavin containing monooxygenase 3, transcript variant 2	6.5978975	0.01010101
HOXA7	Homeobox A7	4.861581	0.02020202
FMO2	Flavin containing monooxygenase 2 (non-functional), transcript variant 1	4.451478	0.02020202
CYP2C8	Cytochrome P450, family 2, subfamily C, polypeptide 8, transcript variant 1	3.8967078	0.01010101
IGF2	Insulin-like growth factor 2, transcript variant 1	3.6979346	0.01010101
SLC14A2-AS1	SLC14A2 antisense RNA 1, long non-coding RNA	3.484851	0.01010101
Clone– BU567832	AGENCOURT_10399047 NIH_MGC_82 cDNA clone IMAGE:6614537 5′	3.0842583	0.02020202
GINS1	GINS complex subunit 1 (Psf1 homolog)	2.826527	0.04040404
MCF2L	cDNA FLJ12122 fis, clone MAMMA1000129	2.80219	0.01010101
ZNF611	zinc finger protein 611	2.2500026	0.030303031
PLCD1	Phospholipase C, delta 1, transcript variant 2	−2.3632836	0.02020202
FBXL22	F-box and leucine-rich repeat protein 22	−2.7893722	0.030303031
EMID1	EMI domain containing 1, transcript variant 1	−4.5672774	0.030303031
LYVE1	Lymphatic vessel endothelial hyaluronan receptor 1	−5.93999	0.030303031
CSTA	Cystatin A (stefin A)	−6.0303144	0.02020202
UBE2QL1	Ubiquitin-conjugating enzyme E2Q family-like 1	−6.751078	0.04040404
DES	Desmin	−7.8715506	0.01010101
INSL3	Insulin-like 3 (Leydig cell), transcript variant 2	−25.868898	0.01010101
INSL3	Insulin-like 3 (Leydig cell), transcript variant 2	−69.227425	0

FC(abs) – absolute fold change, advanced vs stage 1 aGCT

*p*(Corr) – corrected *p* value.

**Figure 2:**
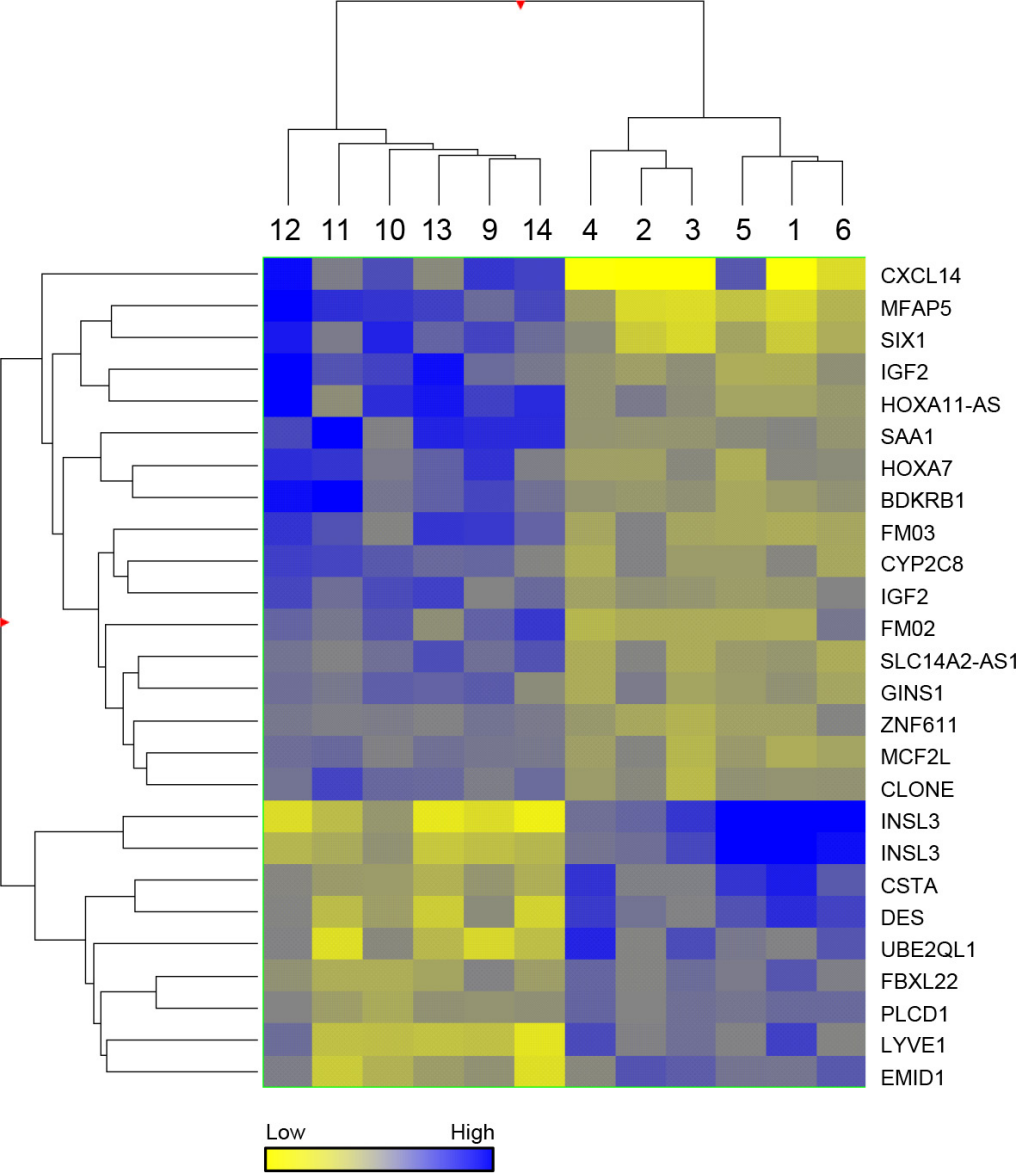
Heat map Hierarchical analysis clustered by normalised intensity values of the 26 statistically significant probes between stage 1 and stage 3 aGCT, using a Euclidean similarity measure and Ward's linkage rule.

**Figure 3:**
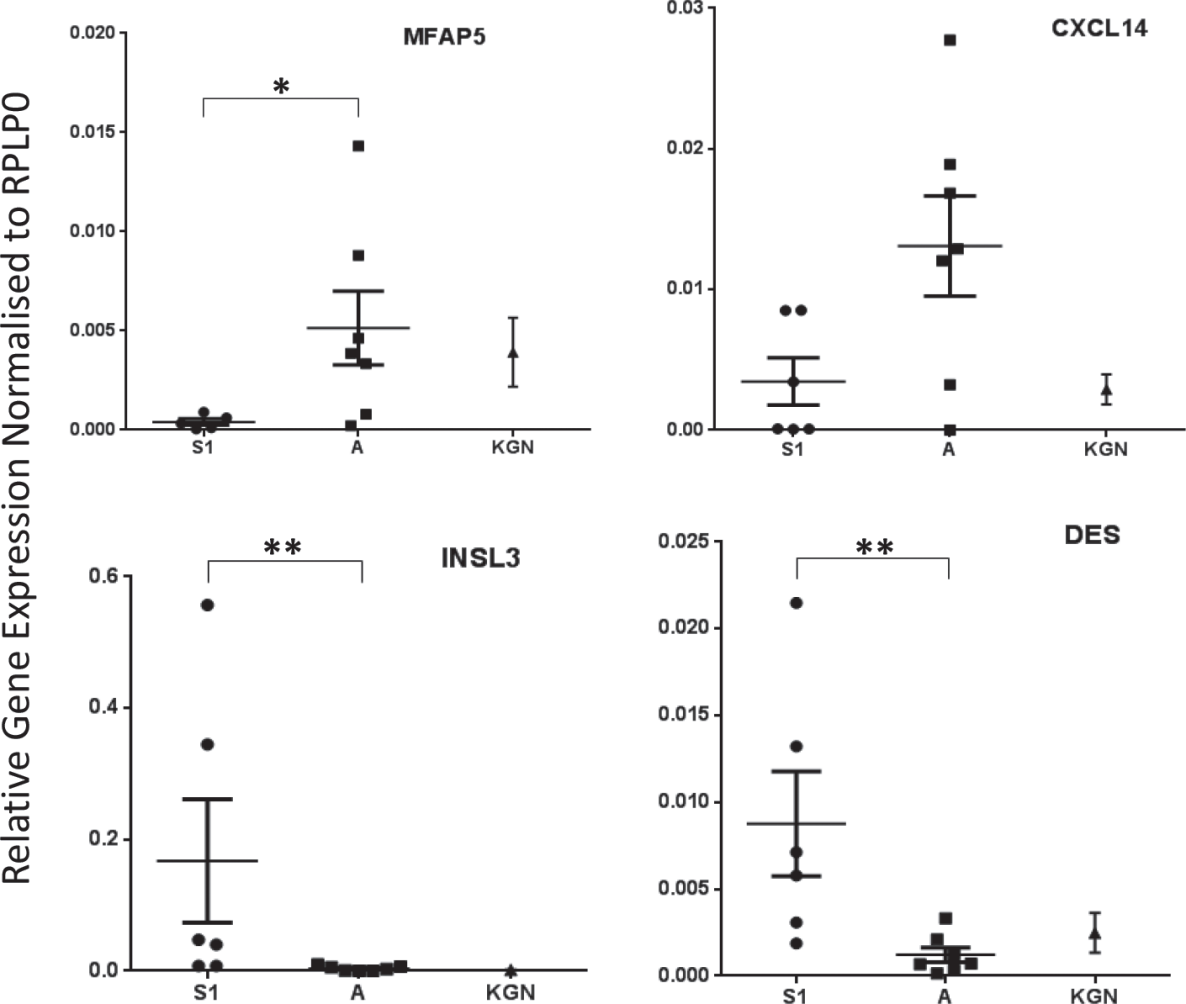
Scatter plots representing relative gene expression of MFAP5 (*p* = 0.0303), CXCL14 (*p* = 0.1014), INSL3 (*p* = 0.0047) and DES (*p* = 0.0082) in stage 1 vs stage 3 aGCT samples The mean +/− standard error of the mean from 3 independent experiments is shown. Samples were normalised to RPLP0 gene expression and the non-parametric Mann-Whitney test was performed. KGN data is also shown.

Unsupervised hierarchical clustering of the 24 genes is presented as a Heat Map which shows clear discrimination of the two groups (Figure 2). The observed changes were assessed for 4 genes, selected on the basis of their fold change and *p*-value, by quantitative RT-PCR (Figure 3) using an overlapping but non-identical group of tumors (Table 1): microfibrillar-associated protein 5 (MFAP5) which was significantly more highly expressed in the stage 3 group; and insulin-like 3 (INSL3) and desmin (DES) which were significantly more abundant in the stage 1 aGCT. For the orphan cytokine CXCL14, the grouped data did not achieve significance reflecting the heterogeneity in the observed levels within each group. The results are presented as a scatterplot; the levels observed in the KGN cell line are also indicated (Figure 3).

**Figure 4:**
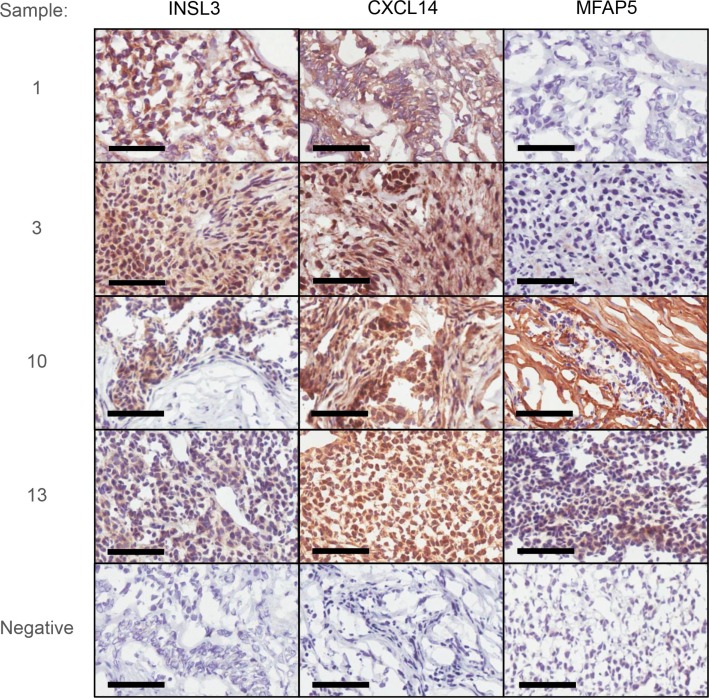
Immunohistochemical examination of INSL3, CXCL14 and MFAP5 in two stage 1 (1 and 3) and two stage 3 aGCT (10 and 13) Negative controls are shown as inserts for each sample. Bars correspond to 60 μ.

The expression of 3 of these genes, (INSL3, CXCL14 and MFAP5) was examined at the protein level using immunohistochemistry (Figure 4); the results, although semi-quantitative are consistent with the relative abundances observed at a RNA level: stage 1 vs stage 3 aGCT. Of more importance however, is that the immunohistochemistry confirms that these genes are indeed expressed in the tumor cells *per se*.

The transcriptome from the KGN cells when compared with the stage 3 aGCT showed substantial differences. In an analysis of all 12 aGCT compared to the KGN cells, 3674 entities differed by ≥ 2-fold at a *p*-value of ≤ 0.05 and passed a Westfall Young Permutative multiple correction test. Of the 24 genes that differ between the stage 1 and advanced aGCT, only 5 differed: SIX1 (SIX homeobox 1), BDKRB1 (bradykinin receptor B1), FMO3 (flavin-containing monooxygenases 3) and GINS1(GINS subunit 1) were increased in the KGN cells when compared to all of the aGCT, whereas MCF2L was significantly lower in the KGN cells. In that the KGN cell line is derived from a very aggressive aGCT [3], a comparison with the transcriptome of the stage 3 aGCT alone may be seen as more appropriate. That comparison again shows a substantial differential expression with 4369 entities (≥ 2-fold; *p*-value of ≤ 0.05, after a Westfall Young Permutative multiple correction test). Of the 24 genes that differ by stage, 15 differed when the stage 3 aGCT and KGN transcriptomes are compared. PLCD1 (a member of the phospholipase C family), EMID1 (EMI domain containing 1), CSTA (cystatin A) and INSL3 (Figure 3), which are down in the stage 3 when compared to the stage 1 aGCT, are further significantly down in the KGN cells. Curiously, of the genes that are increased in the stage 3 aGCT and therefore might be expected to be further increased in the KGN cells, CXCL14 (Figure 3), MFAP5 (Figure 3), CYP2C8 (cytochrome P450 2C8), IGF2 (insulin-like growth factor 2), MCF2L (MCF.2 cell-line -derived transforming sequence-like) and ZNF611 (zinc finger protein 611) are lower in the KGN cells than the aGCT, whereas SIX1, BDKRB1, FMO3 and GINS1 expression is further increased in the KGN cells.

**Figure 5:**
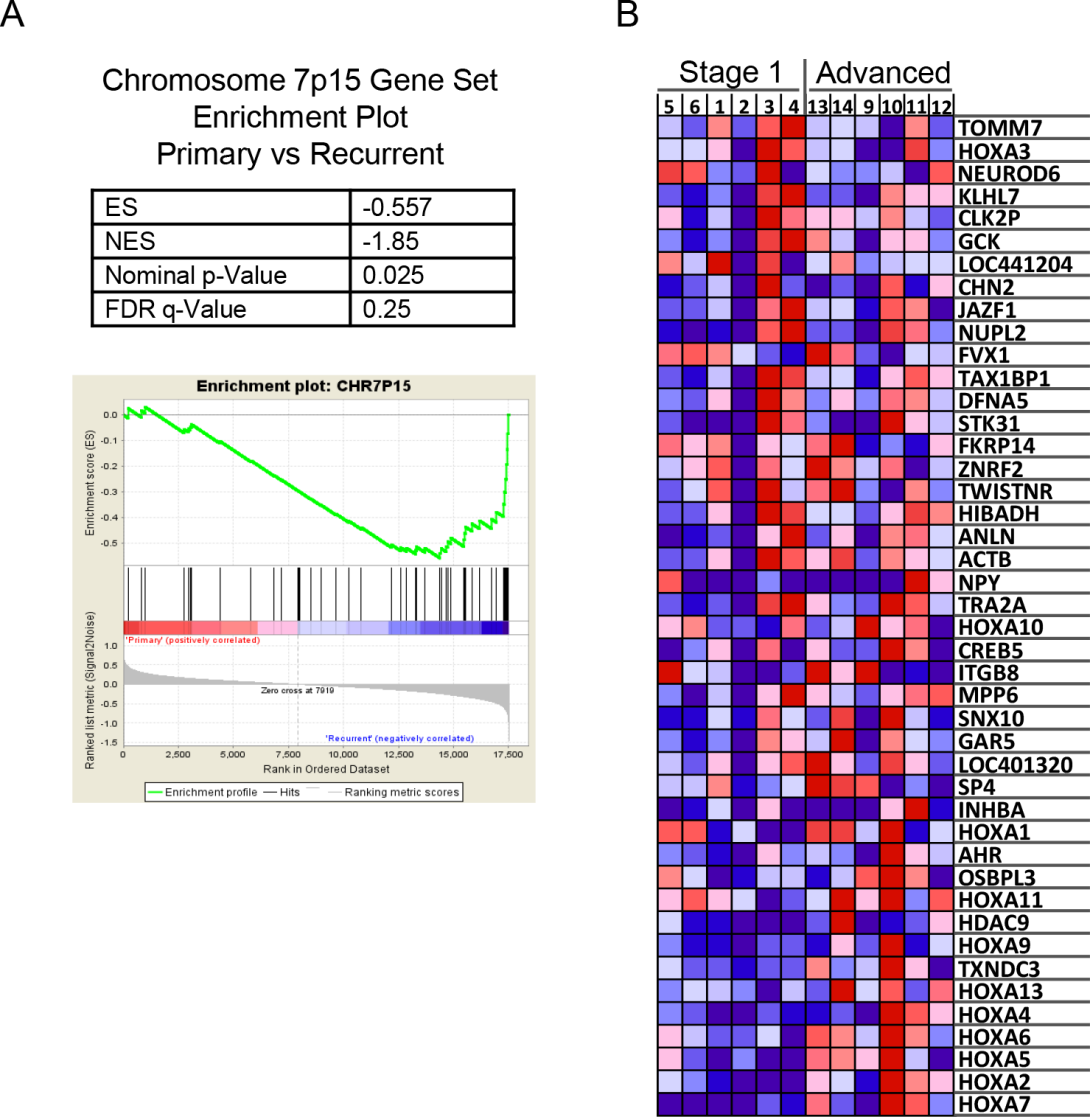
Gene set enrichment analysis (GSEA) from the microarray data comparing stage 1 with stage 3 aGCT showing enrichment of genes clustered on chromosome 7p15 (**A**) GSEA Enrichment plot shows values for the Enrichment Score (ES), Normalized Enrichment Score (NES), nominal *P*-value and False Discovery Rate *q*-Value (FDR-*q* value). (**B**) GSEA generated heatmap for highly enriched genes on Chromosome 7p15 in stage 1 compared to stage 3 aGCT.

Two other methods were used to interrogate the microarray data. The ontogeny of the genes identified as differing between the two groups of genes was examined using the MetaCore^™^ software analysis suite, however no pathways or processes were identified that achieved significance. The microarray data was also analysed using the Gene Set Enrichment Analysis (GSEA) method [12]. The GSEA software also performs an unsupervised hierarchical Heat Map that validated many of the genes identified using the Genespring software, and also similarly shows clear discrimination of the two groups when grouping the top 50 features for each tumor type (Supplementary Figure 1). The microarray datasets for stage 1 and stage 3 aGCT were analysed against the curated MSigDB v4.1 genesets in order to determine whether an *a priori* defined set of genes show statistically significant, concordant differences between stage 1 and stage 3 aGCT. Using this method, we identified a significant enrichment of genes located on chromosome 7p15 (Figure 5, Supplementary Table 2), with an enrichment score (ES) of −0.557 (*p* = 0.025) in stage 1 aGCT vs stage 3 aGCT (Figure 5A). This region includes the homeobox A (HOXA) gene locus. A graphical view of the over-represented and overexpressed genes located on chromosome 7p15 in stage 3 aGCT compared to stage 1 aGCT is shown in Figure 5B.

## DISCUSSION

Comparison of the gene expression profiles revealed a relatively small number of genes that differ between the stage 1 and the advanced, stage 3 aGCT. This relative homogeneity across stage in part reflects the presumption of shared aetiology with respect to cell type of origin and a shared initiating event, the FOXL2 C134W mutation. This homogeneity is consistent with observations in other studies of expression of specific genes in aGCT [13–17] and indeed also with the observations that aGCT exhibit relative genomic stability when compared to epithelial ovarian cancers [2]. Although the tumor classification is robust with respect to aGCT, i.e. all contain the FOXL2 C134W mutation, the designation of stage could potentially be problematic in that a stage 1 aGCT could, for instance, be an advanced tumor caught early. The robust distinction between the groups reflected in the 24 genes identified (Figure 2) however argues strongly for the validity of the prospective classification used. Attempts to segregate the data using other comparisons, e.g. age, menopausal status, did not reveal distinct patterns of expression.

Of the 8 genes whose expression is significantly diminished in the stage 3 aGCT, INSL3 stands out as a robust discriminator being ∼70-fold higher in stage 1 disease with no overlap between groups (Figure 3). INSL3 is a member of the insulin-like hormone superfamily that is predominantly expressed in gonadal tissues. Its actions are mediated by the relaxin-insulin-like family peptide receptor 2 (RXFP2). Although RXFP2 expression appears higher in the stage 1 aGCT this was not significant (data not shown). INSL3 is expressed in the adult ovary primarily in theca and luteal granulosa cells where it is thought to have a role in maintenance of steroidogenesis [18]. INSL3 expression has recently been shown to be regulated in Leydig cells by COUP-TFII [19] which is abundantly expressed in aGCT [17]. INSL3 expression is inhibited by the bone morphogenetic proteins [18]; this diminished expression with stage 3 disease may be a bystander effect or perhaps reflect activation of the BMP-SMAD signalling pathway in advanced aGCT.

Desmin expression is also significantly reduced in the stage 3 aGCT being 7-fold higher in stage 1 disease. Desmin is a class III intermediate filament usually associated with muscle, however it has been observed to be expressed strongly in a range of tumors unrelated to muscle [20]; the significance of the decrease in expression in aGCT with advanced disease is not clear. Similarly UBE2QL1 – ubiquitin-conjugating enzyme E2Q family-like 1 is down-regulated in the stage 3 aGCT. There is a very limited literature for UBE2QL1 in malignancy; however one study [21] ascribes a tumor suppressor function to UBE2QL1 which could be seen as consistent with its down-regulation in the context of more aggressive malignancy. Similarly, PLCD1, which encodes a member of the phospholipase C family, has higher expression in the stage 1 tumors consistent with reports in several tumor types that it is a tumor suppressor gene [22]. EMID1 is decreased in the stage 3 aGCT but information about its function is limited.

Other genes whose expression was higher in the stage 1 aGCT exhibited more modest differences. F-box and leucine-rich repeat protein 22 (FBXL22) is a member of the F-box gene family. It interacts with S-phase kinase associated protein 1A and cullin to form a complex with ubiquitin-ligase activity [23]. LYVE1 (lymphatic vessel hyaluranon receptor-1) expression is ∼6-fold higher in the stage 1 aGCT. LYVE1 is a type 1 integral membrane glycoprotein predominantly expressed in lymphatic vessels [24]; indeed it has been extensively used as an immunohistochemical marker of lymphatics [25]. The decreased levels may be consistent with a less organised tissue structure with advancing malignancy. The CSTA (cystatin A) gene is also ∼6-fold more abundant in the stage 1 tumors. This gene encodes stefin A, a cysteine protease inhibitor which is thought to primarily inhibit cathepsin B [26]. Cathepsin B expression, although abundant from the microarray analyses, does not differ between the two stages. Loss of CSTA expression is associated with progression of ductal breast cancer *in situ* to invasive breast cancer [26], presumptively via increased cathepsin B activity which raises the possibility that cathepsin B may be a relevant therapeutic target in aGCT.

Of the 16 genes whose expression was increased with advanced disease, CXCL14 shows the greatest increase. CXCL14 is an orphan member of the Cys-x-Cys subfamily of cytokines which, although chemotactic for cells of the monocyte/macrophage lineage, appears not to be required for their normal function [27]. CXCL14 expression is markedly up-regulated (∼40-fold) in the stage 3 aGCT in the microarray analysis but not significant in the RT-PCR (Figure 3) reflecting a striking hererogeneity of expression, particularly in the stage 3 aGCT. CXCL14 has been associated with both favourable and unfavourable outcomes depending on tumor type [28]. Augsten et al. [27] argue that pro-tumoral effects of CXL14 reflect overexpression in cancer-associated fibroblasts however immunohistochemical staining of the aGCT (Figure 4) shows clear expression in the tumor cells. A possible explanation of this apparent dichotomy may lie with the stromal origin of granulosa cells. Riesten et al. [29] have recently identified CXCL14 as a key element in a gene expression signature that predicts outcome in advanced epithelial ovarian cancer. Fibroblast activating protein (FAP) which is abundantly expressed in both groups of aGCT is also part of that signature. Like CXCL14, FAP expression has been associated with cancer-associated fibroblasts. FAP shows ∼4 fold increase in the stage 3 aGCT but this is not significant after correction for FDR (false discovery rate). It is however of interest, being an emerging therapeutic target with considerable specificity [30]. FAP is a cell surface glycoprotein with dipeptidyl peptidase activity whose expression is normally restricted to fibroblasts in healing wounds [31]. Some years ago we were surprised to find that it was also expressed by the normal human ovary [32]. Whilst its expression in normal ovary may reflect the “wound healing response” associated with ovulation, it raises the possibility that FAP is a product of GC, which as noted are stromal in origin. Conversely, FAP expression is not observed in the KGN cells which may argue that it is not a feature of advanced aGCT per se. Secretion by activated stromal fibroblasts of FAP has also been associated with epithelial ovarian cancer cell proliferation, migration and invasion [33].

MFAP5 showed the second greatest (∼26)-fold increase. MFAP5 is a microfibrial-associated glycoprotein which predicts poor survival and chemoresistance in patients with advanced high-grade serous epithelial ovarian cancer [34]. Of the other genes that are significantly upregulated with advanced disease, SAA1 (serum amyloid A1) is part of the family of highly homologous acute-phase proteins that have been associated with tumor progression and reduced survival in a range of cancers [35]. Of the other members of this family, SAA2, S100P (serum 100 calcium binding protein P), S100B, S100A7, S100A3, S100A1, S100A3 and S100PBP (S100P binding protein) were identified on the microarray but did not differ between groups.

SIXl is a homeobox gene which has a well characterised role in development [36]; it has also been found to be upregulated in a number of solid tumors which correlates with a worse prognosis. SIX1 expression was further increased in the KGN cells suggesting an association with increasing malignancy. SIXl increases cyclin D1 expression in solid tumors [36], however cyclin D1 levels as determined from the microarray data did not differ by stage in the aGCT. Cyclin D2 plays a critical role in granulosa cell proliferation and has increased expression in GCT [13, 37]; however, although abundantly expressed being ∼10 fold higher than cyclin D1, cyclin D2 expression also did not differ by stage.

FMO2 and FMO3 encode flavin-containing monooxygenases whose expression was also increased in advanced disease. They are found in a cluster with the other FMO genes: FMO1, and FMO4, although neither of these genes differ by stage. These NADPH-dependent flavoenzymes catalyse the oxidation of numerous drugs and xenobiotics. FMO3 is predominately expressed in the liver where it plays a role in the metabolism of xenobiotics including a number of anti-cancer drugs [38]. Aside from hepatic tumors, there is little work on the role of FMO3 in other tissues or tumors. Curiously, it is expressed in the Fallopian tubal epithelium [39]. One might speculate that FMO3 upregulation might be associated with resistance to chemotherapy. FMO3, but not FMO2, is one of the 4 genes whose expression is further increased in the KGN cells. CYP2C8 whose expression is also increased in the stage 3 aGCT, is a cytochrome P450, known to metabolise many xenobiotics including paclitaxel [40]. SLC14A2 (solute family 14, member A) is a urea transporter normally expressed in the renal epithelium has not previously been associated with malignancy but may have a role in the efflux of toxic metabolites from the tumor cells.

HOXAll-ASl is a non-coding anti-sense transcript directed at the homeotic HOXAll gene whose biology has been described in the human endometrium [41]. Epigenetic silencing of HOXA11 has been observed in a number of tumors including ovarian cancer [42]; it is associated with a worse prognosis and/or chemotherapy resistance. HOXA11 expression appeared low but did not differ between groups. This contrasts with the HOXA7 homeobox gene whose expression is increased with advanced disease. We observed a significant enrichment of genes located on chromosome 7p15 using GSEA. Significantly, this locus contains many of the HOX genes, including HOXA7. HOXA7 expression has previously been reported in normal granulosa cells and in KGN cells where it regulates expression of the epidermal growth factor receptor [43], however there was no evidence of a change in the expression of this receptor. Knock-down of HOXA7 expression in KGN cells has been reported to decrease cell proliferation, again arguing for an active role for HOXA7 in GCT tumorigenesis [43].

MCF2L, which encodes a guanine nucleotide exchange factor, is expressed in articular chondrocytes [44]; the significance of its increased expression in stage 3 aGCT is unclear. BDKRB1 is synthesised *de novo* following tissue injury. Its expression has been reported to be upregulated in a range of tumors including another stromal tumor, chondrosarcoma [45], but its role in malignancy is unclear. The ligands for this receptor, bradykinin and kallidin, are known to induce angiogenesis, cell migration and metastasis [46] so the increased expression could relate to cells other than the tumor cells (e.g. inflammatory cells, the vasculature), however expression in the KGN cells supports a tumoral origin. The GINS complex is associated with the initiation of DNA replication in yeast and Xenopus. GINS1, also known as PSF1, has been associated with high proliferative activity [47] and indeed its expression is also further increased in the KGN cell line.

Increased expression of IGF2 is seen in the stage 3 aGCT, as in a broad range of tumors, where it is associated with increasing malignancy [48]; its mitogenic properties have been extensively characterised. We have previously described IGF1 and 2 expression in a mixed group of GCT which was heterogenous but we did not analyse this by stage or type [15]. The increased expression in the stage 3 aGCT of ZNF611, a member of the large family of zinc finger containing transcription factors located on chromosome 19, and the clone BU567832, is of uncertain significance, neither having previously been associated with malignancy. The clone BU567832 localises to an intrageneic region of chromosome 18; whilst transcripts have been reported in other tissues, the nature of the RNA detected remains to be determined.

The previous aGCT transcriptomic study by Benayoun et al. [9] using predominantly stage 1 tumors (9 of 10 aGCT) compared to two granulosa cell samples obtained at IVF identified changes in the expression of FOXL2 regulated genes consistent with the presence of the FOXL2 p.Cys134Trp mutation. In the current study, all tumors have this mutation so this same pattern of enrichment, as expected, was not observed. Rosario et al. [11] applied transcriptomic analysis to the KGN cells and another human GCT-derived cell line, COV434, which in contrast to the KGN cells neither expresses FOXL2 nor contains the p.Cys134Trp mutation, consistent with it having been derived from a juvenile GCT [3]. They identified a number of differences between the two but the genes were not amongst those identified in the current study. The marked difference between the aGCT transcriptome and that of the KGN cells argues for some caution in extrapolating findings *in vitro* using KGN cells to aGCT.

Of the genes decreased in advanced disease, several may reflect a loss of the differentiated state eg INSL3, desmin; the others do not clearly reflect specific pathways relevant to the advanced malignancy. Similarly, of the genes upregulated in advanced disease many have been associated previously with advanced malignancy in other tumor types; those that are further increased in the KGN cells, particularly SIX1 and GNS1 which have established associations with malignancy and proliferation, respectively, may represent drivers of the neoplastic process.

In seeking to understand the changes in gene expression we applied pathway and process analyses but this was not revealing, likely reflecting the relatively small number of genes that differed by stage. Gene set enrichment analysis highlighted over expression of genes on chromosome 7p15 in stage 3 aGCT. This finding is consistent with a report investigating the prognostic significance of chromosomal imbalances detected using CGH for GCT [49] which reported gain of 7p15-p21 to be a feature in some GCT samples. Other studies using cytogenetics or CGH have variably identified trisomy of chromosomes 12 and 14 in approximately one third of cases [10, 50] with a lesser frequency in several other chromosomes/locii. Increased HOX gene mRNA levels may also simply reflect co-ordinate increased expression of this locus as can be observed at certain stages of development. Our analysis demonstrates a rather compelling association of the HOXA locus on 7p15 with stage in that the relative expression of the locus taken as a whole clearly segregates with stage. One might argue that an amplification of the HOX gene locus represents a strong candidate marker for advanced disease; whether this has prognostic value i.e. can predict recurrent or aggressive disease will require a prospective study.

Although the majority of stage 1 aGCT are cured with surgery, advanced disease represents a significant therapeutic challenge. These studies identifies a panel of genes that differ between stage 1 and stage 3 aGCT; in some cases they robustly discriminate the stages (Figure 3). It remains to be established whether these differences can be used to establish prognosis. Thus confirmation in an independent cohort and a prospective study will ultimately be required. The molecular basis of these changes in gene expression remains to be determined; although a large number of known and putative oncogenes have been examined in aGCT [1], an unbiased mutation screen, aside from the study of Shah et al. [2] in which 4 aGCT were examined, has not been reported. The expression profiles do however identify several overexpressed genes in both stage 1 and stage 3 aGCT, or just the stage 3 aGCT, which warrant further study as potential therapeutic targets. Some, such as FAP appear relatively stromal cell specific, whilst others are emerging targets in other tumor types.

## METHODS

RNA was isolated from 6 stage 1 aGCT and 6 stage 3 aGCT collected sequentially and predominantly at our institution [3, 6]. Stage is defined according to the FIGO (International Federation of Gynecology and Obstetrics) criteria used for ovarian cancer [51]. The stage 3 aGCT were all collected at a surgery subsequent to their initial surgery and may thus be interpreted as either a recurrence or progression of a known aGCT. The GCT-derived cell line, KGN which is heterozygous for the FOXL2 mutation has been described previously [3, 52].

### Transcriptome profiles

We established transcriptome profiles for the aGCT using the Agilent Whole Human Genome 4 × 44K Expression Microarrays (Agilent Technologies, Santa Clara, CA). Cyanine-3 (Cy3) labeled cRNA was prepared from 200 ng total RNA using the One Color Low Input Quick Amp Labeling Kit (Agilent) followed by RNeasy column purification (Qiagen, Hilden, Germany). Dye incorporation and cRNA yield were checked using the NanoDrop ND-1000 Spectrophotometer (Thermo Scientific, MA). 600 ng of cRNA for each sample was then hybridized onto a separate array for 17 h at 65°C and washed following the manufacturer's instructions. Slides were scanned using an Agilent DNA Microarray Scanner (G5205B) (Agilent) using the one-color scan setting for ‘4 × 44K’ slides. The scanned images were analysed with Feature Extraction Software 9.5.3.1 (Agilent) using default parameters to obtain background-subtracted and spatially detrended processed signal intensities. Data from feature extraction were imported into GeneSpring GX13.1 (Agilent) for analysis. Data was normalised using the quantile normalisation method and tested for significant differences between stage 1 and stage 3 aGCT by performing a moderated *t*-test with the *P* value (≤ 0.05 deemed significant) computed using the asymptotic method. Genes which also had a fold change ≥ 2.0 were then subjected to Westfall Young Permutative multiple testing correction. All data produced was MIAME-compliant.

### Pathway analysis

Of the 17,847 entities identified as expressed in the microarray, 50 of them were found to pass the Moderated *T*-test with a Westfall-Young Permutation method for False Discovery Rate and threshold settings of 1.5 fold and *p*-value of 0. They were then subjected to a pathway analysis with the MetaCore^™^ software analysis suite (Thomson Reuters, New York, NY).

### Gene set enrichment analysis (GSEA)

In addition, the normalised microarray data was analysed using the Gene Set Enrichment Analysis (GSEA) method [12]. The microarray datasets were stratified and assigned phenotypes as stage 1 and stage 3 aGCT. GSEA was then performed for each of the samples using the gene permutation algorithm. Enrichment analysis was performed using the default parameter settings. We compared the gene expression levels from the stage 1 versus the stage 3 aGCT groups and identified the genes that had significantly different expression in the GSEA by using the gene sets from the Molecular Signatures Database (MSigDB v4.1) (www.broadinstitute.org/gsea). The enrichment score (ES) was calculated for each gene set reflecting if the genes in the particular gene set appeared in the top (positive score), in the bottom (negative score), or were randomly distributed (close to zero score). The ranking metric used was the signal-to-noise ratio. Scores were compared with scores calculated from 1,000 randomly permuted gene lists, in order to calculate false discovery rates (FDR) (cut-off at FDR = 0.05). The ES, normalized ES (NES), *p* value, and FDR *q*-value were then used to rank the gene sets. Definitions of these output variables can be found in [12].

### RT-PCR

The comparative Ct (ΔΔCt) method was used to validate four genes. FAM labeled TaqMan Gene Expression assays for CXCL14, MFAP5, INSL3 and DES were purchased along with a FAM labeled RPLP0 probe which was used as an endogenous control. A 10 ul reaction was prepared with 1 × TaqMan universal PCR master mix (Applied Biosystems, Foster City, CA) and diluted cDNA. All PCR reactions were carried out in triplicate in MicroAmp optical 384-well reaction plates (Applied Biosystems). The cycling parameters were initiated by by 2 min at 50°C and 10 min at 95°C, followed by 40 cycles of 15 s at 95°C and 60°C for 1min using the 7900HT fast real-time PCR system (Applied Biosystems).

### Immunohistochemistry

Frozen GCT were blocked with OCT and 4 μm sections were prepared using the cryostat. All incubations and washes were performed at room temperature unless stated otherwise. Frozen GCT sections were fixed in 4% paraformaldehyde for 30 min followed by quenching of endogenous peroxidase using 0.3% hydrogen peroxide/PBS for 30 min. For membrane permeation, slides were incubated in 0.1% triton X-100/PBS for 10 min. Nonspecific binding was blocked by 10% goat serum in 3% BSA for an hour. Incubations with primary antibody, rabbit polyclonal INSL3 (Abcam ab 65981; 1:250), CXCL14 (Abcam ab46010; 1:400) or MFAP5 (Sigma abHPA010552; 1:500) was performed at 4°C overnight. Goat serum was used as a negative control. After PBS washes, slides were incubated with biotinylated goat anti-rabbit secondary antibody (Dako; 1:200) for an hour. VECTORSTAIN® avidin/biotinylated enzyme complex was made up as per manufacturer's instructions, added to sections and incubated for an hour. Staining was visualised by incubation of DAB solution (Dako) for 3 min. Sections were counterstained with hematoxylin, dehydrated with ethanol (70% and 100%) and mounted with DPX. Validation of the antibodies using a positive control tissue is shown in Supplementary Figure 2.

## SUPPLEMENTARY MATERIALS FIGURES AND TABLES


