# Supplementary material for: Transcriptomic analysis of stage 1 versus advanced adult granulosa cell tumors
Source: Oncotarget. 2016 Feb 16;7(12):14207–19. doi: 10.18632/oncotarget.7422 (PMC4924709; doi:10.18632/oncotarget.7422)
Supplement: Supplementary file 1 [file oncotarget-07-14207-s001.pdf]

Transcriptomic analysis of stage 1 versus advanced adult granulosa cell tumors

Supplementary Materials

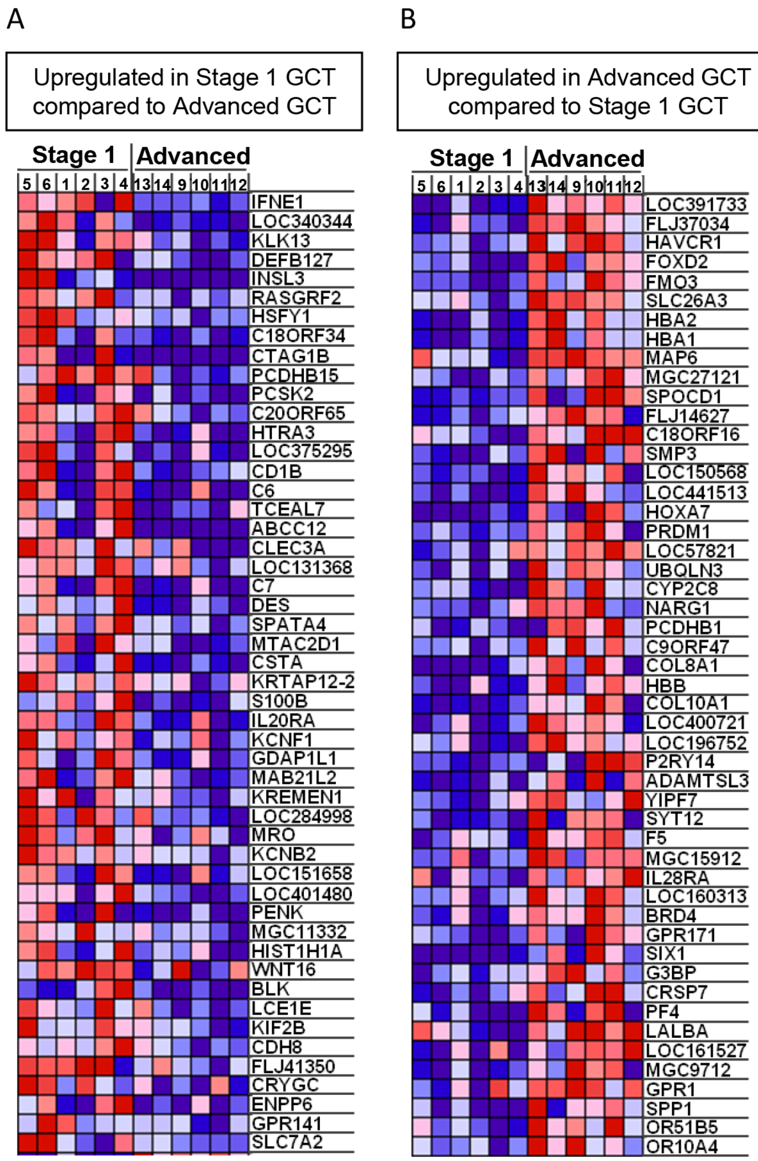

Supplementary Figure S1: Expression profile of microarray data by GSEA. (**A**) Genes upregulated in stage 1 when compared to advanced GCT. (**B**) Genes upregulated in advanced GCT when compared to stage 1. Heat map represents the top 50 features for each phenotype.

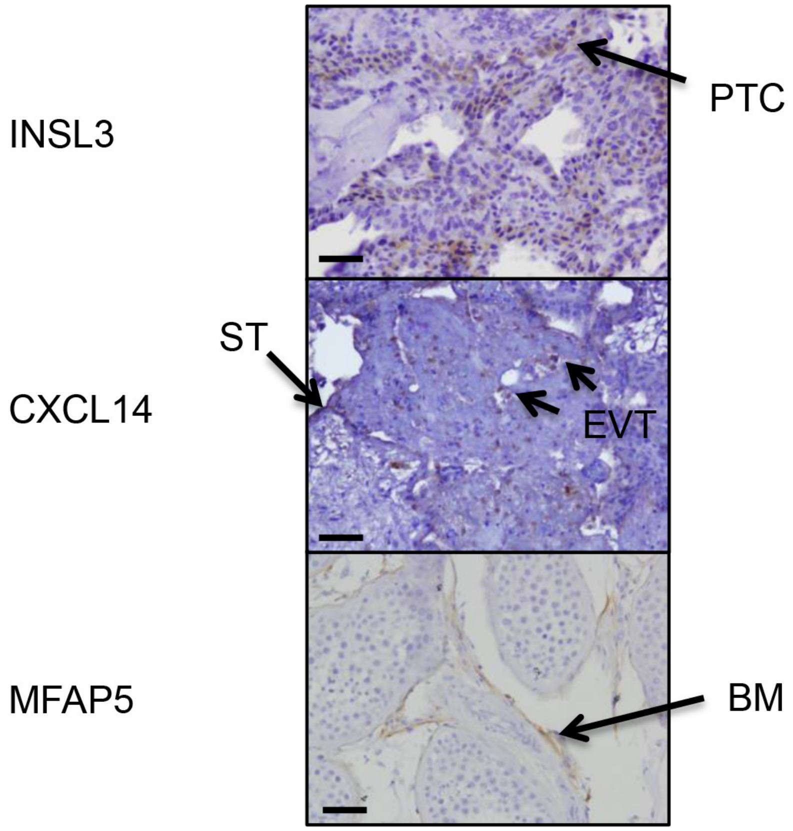

Supplementary Figure S2: Positive control for immunohistochemistry for INSL3, CXCL14 and MFAP5 in papillary thyroid cancer, human placenta and testis, respectively. Positive staining of INSL3 is seen in papillary thyroid cancer cells (PTC). CXCL14 is expressed in syncytiotrophoblast (ST) and extravillous trophoblast (EVT) in human placenta. MFAP5 is expressed in the basement membrane (BM) in human testis. Bars correspond to 100 µm.

Supplementary Table S1: Full list of the 26 gene probes

Probe Name	Gene Symbol	Description	Sequence
A_24_P919850	BDKRB1	Bradykinin receptor B1	TTCATCCCATAGGAAAGAAATCTTCCAACTTT TCTGGCGGAATTAAAACAGCATTGAACC
A_32_P118325	Clone – BU567832	AGENCOURT_10399047 NIH_MGC_82 cDNA clone IMAGE:6614537 5′	ATTAATTCTGACTAAATGGCATTAAAGGAGGTC TGAGGAATTTGCATGTCACATTCTGGA
A_23_P170233	CSTA	Cystatin A (stefin A)	AACTGGCTACTGAGTCATGATCCTTGCTGATAA ATATAACCATCAATAAAGAAGCATTCT
A_23_P213745	CXCL14	Chemokine (C-X-C motif) ligand 14	GTGGTACAACGCCTGGAACGAGAAGCGCAGGG TCTACGAAGAATAGGGTGAAAAACCTCA
A_23_P161368	CYP2C8	Cytochrome P450, family 2, subfamily C, polypeptide 8, transcript variant 1	AAACTTGGTTGGCACTGTAGCTGATCTATTTGT TGCTGGAACAGAGACAACAAGCACCAC
A_23_P90710	DES	Desmin	CCAGCCCAGGGTGGACTTAGAAAGCAGGGGC TACAAGAGGGAATCCCCGAAGGTGCTGGA
A_24_P14634	EMID1	EMI domain containing 1, transcript variant 1	GAGGCAGGCCTTCAAGGAGGGATAGAGGTAC AAGGCTTCGTCTCATCTGCTGTCTGAGCA
A_32_P334875	FBXL22	F-box and leucine-rich repeat protein 22	AAGAGTGCCTTCCAGAGAAGCATCTGCAGCCG GCACGAGAGCCTGGTCAATGATTTCCTC
A_23_P355295	FMO2	Flavin containing monooxygenase 2 (non-functional), transcript variant 1	CCCTGAAGGATTCATCTAATTTCTCAGNTCTTTT CTGTTGAAAATCCTGGGCCTTCTTG
A_23_P155596	FMO3	Flavin containing monooxygenase 3, transcript variant 2	TCGGGAGACTTCAGAAGCCTTGCTTCTTTTTCC ATTGGCTGAAGCTCTTTGCAATTCCTA
A_23_P210853	GINS1	GINS complex subunit 1 (Psf1 homolog)	TCTTGCCAAATGCATTACGATTTCACATGGCTG CTGAAGAAATGGAGTGGTTTAATAATT
A_24_P280983	HOXA11-AS	HOXA11 antisense RNA	TGAGCCTTACGCTTCTTTCCACAGCCTTTGCAG GCGGAATATCGGAATAAAGTGGGTCCA
A_23_P70968	HOXA7	Homeobox A7	GAAAAGCGTCTTTAAGAGACTCACTGGTTTTA CTTACAAAAATGGGAAAAATAAAAGAAA
A_23_P150609	IGF2	Insulin-like growth factor 2, transcript variant 1	CTCAACTCAGCTCCTTTAACGCTAATATTTCCGG CAAAATCCCATGCTTGGGTTTTGTCT
A_23_P421379	IGF2	Insulin-like growth factor 2, transcript variant 1	TGCTTCCGGACAACTTCCCCAGATACCCCGTGG GCAAGTTCTTCCAATATGACACCTGGA
A_23_P209006	INSL3	Insulin-like 3 (Leydig cell), transcript variant 2	CCTGCATGTGTAACACCCCTTCTTGCTGTCTCTT AGTAAATAAACGACCCAAAGCAGCTT
A_23_P309281	INSL3	Insulin-like 3 (Leydig cell), transcript variant 2	CAACCCTGCACGCTACTGCTGCCTCAGTGGCTG TACCCAACAAGACCTGCTGACCCTCTG
A_23_P150457	LYVE1	Lymphatic vessel endothelial hyaluronan receptor 1	CTCTGTAAAGCTAAAATAAAGAAATAGAACAAG GCTGAGGATACGACAGTACACTGTCAG
A_23_P99496	MCF2L	cDNA FLJ12122 fis, clone MAMMA1000129	GCTTT TCTAAGAATGGAGTACTCGT T T TCAAG AGATTTGTCCTAATTATATTTTCCAGCG
A_23_P87700	MFAP5	Microfibrillar associated protein 5, transcript variant 1	TGAGAGGAAGGAGGATCTCCTTCTTCTCCAACC ATTGACAGCTAACCCTTAGACAGTATT
A_23_P80739	PLCD1	Phospholipase C, delta 1, transcript variant 2	CAGCCTCTTGCTCAGAGCTAGGCCCCCAAATTG CCTTCAGCCCTAACATAGTGTCTGCTG

A_24_P335092	SAA1	Serum amyloid A1, transcript variant 1	CAGAAGCGATCAGCGATGCCAGAGAGAATATCCAGAGATTCTTTGGCCATGGTGCGGAGG
A_23_P76914	SIX1	SIX homeobox 1	AGCTTGTTTCTGGAGTTGTTTGCGCATAAAGGAATGGTGGACTTTCACAAATATCTTTTT
A_32_P179370	SLC14A2–AS1	SLC14A2 antisense RNA 1, long non-coding RNA	GTCTTATGAATCTCTACTTAGTTGACCACAAGTAGTAAGCAAGAAACAATCCTGTAAAGA
A_32_P85131	UBE2QL1	Ubiquitin-conjugating enzyme E2Q family-like 1	ACGCATGTGGCATAAATCAACAGGAAAGAAATGTTTACCACTGGAAGATGGTGAGTGCAT
A_24_P205154	ZNF611	zinc finger protein 611	TAGAGATTGAGGACGATTGAAAGATTGGGGAGAAGGAGCAACAAGAGGTTAAATAAGGTT

Supplementary Table S2: CHR7P15 gene enrichment list

GENE SYMBOL	GENE_TITLE	RANK IN GENE LIST	RANK METRIC SCORE	RUNNING ES	CORE ENRICHMENT
TOMM7	translocase of outer mitochondrial membrane 7 homolog (yeast)	209	0.454372376	0.025900656	No
HOXA3	homeobox A3	811	0.310176522	0.01735114	No
NEUROD6	neurogenic differentiation 6	996	0.288120657	0.030829143	No
KLHL7	kelch-like 7 (Drosophila)	2770	0.165355176	−0.056862973	No
CLK2P	CDC-like kinase 2, pseudogene	2947	0.156909823	−0.053860668	No
GCK	glucokinase (hexokinase 4, maturity onset diabetes of the young 2)	3028	0.153573558	−0.04564217	No
LOC441204	-	3095	0.150824398	−0.036851525	No
CHN2	chimerin (chimaerin) 2	4384	0.103601053	−0.101932384	No
JAZF1	JAZF zinc finger 1	5805	0.060347509	−0.17817198	No
NUPL2	nucleoporin like 2	6852	0.030363288	−0.23550566	No
EVX1	eve, even-skipped homeobox homolog 1 (Drosophila)	7188	0.020422678	−0.25297633	No
TAX1BP1	Tax1 (human T-cell leukemia virus type I) binding protein 1	7924	−1.30E−04	−0.29503042	No
DFNA5	deafness, autosomal dominant 5	7958	−0.001305739	−0.29681024	No
STK31	serine/threonine kinase 31	8023	−0.00343373	−0.3001869	No
FKBP14	FK506 binding protein 14, 22 kDa	8523	−0.017971398	−0.32724774	No
ZNRF2	zinc and ring finger 2	8975	−0.031071296	−0.3504699	No
TWISTNB	TWIST neighbor	9669	−0.051021084	−0.38587958	No
HIBADH	3-hydroxyisobutyrate dehydrogenase	10257	−0.068437472	−0.4137715	No
ANLN	anillin, actin binding protein	10810	−0.085664749	−0.43822485	No
ACTB	actin, beta	12177	−0.134153873	−0.5052238	No
NPY	neuropeptide Y	12599	−0.150776401	−0.5167542	No
TRA2A	-	12869	−0.162419781	−0.5186153	No
HOXA10	homeobox A10	13285	−0.182385162	−0.5271684	No
CREB5	cAMP responsive element binding protein 5	13315	−0.183787122	−0.5135135	No
ITGB8	integrin, beta 8	13703	−0.203251779	−0.5187254	No
MPP6	membrane protein, palmitoylated 6 (MAGUK p55 subfamily member 6)	14386	−0.240703881	−0.53769964	Yes
SNX10	sorting nexin 10	14435	−0.243814513	−0.52013016	Yes
GARS	glycyl-tRNA synthetase	14679	−0.259360343	−0.5124253	Yes
LOC401320	-	14733	−0.263116896	−0.49353355	Yes
SP4	Sp4 transcription factor	14873	−0.27469191	−0.47859913	Yes
INHBA	inhibin, beta A (activin A, activin AB alpha polypeptide)	15472	−0.320280552	−0.486135	Yes
HOXA1	homeobox A1	15497	−0.321670651	−0.46070436	Yes
AHR	aryl hydrocarbon receptor	15556	−0.326089591	−0.43685135	Yes
OSBPL3	oxysterol binding protein-like 3	15839	−0.350884646	−0.423752	Yes
HOXA11	homeobox A11	16178	−0.380331814	−0.41140383	Yes
HDAC9	histone deacetylase 9	16717	−0.443397701	−0.40524673	Yes
HOXA9	homeobox A9	16948	−0.48647207	−0.37787312	Yes
TXNDC3	thioredoxin domain containing 3 (spermatozoa)	17261	−0.58169663	−0.34725758	Yes
HOXA13	homeobox A13	17305	−0.607286334	−0.29911453	Yes
HOXA4	homeobox A4	17379	−0.652338684	−0.24893428	Yes
HOXA6	homeobox A6	17420	−0.700236738	−0.19287416	Yes
HOXA5	homeobox A5	17433	−0.717161775	−0.13380124	Yes
HOXA2	homeobox A2	17465	−0.765490949	−0.07178852	Yes
HOXA7	homeobox A7	17500	−0.895857275	9.16E–04	Yes
